# Meal timing, meal frequency, and breakfast skipping in adult individuals with type 1 diabetes – associations with glycaemic control

**DOI:** 10.1038/s41598-019-56541-5

**Published:** 2019-12-27

**Authors:** Aila J. Ahola, Stefan Mutter, Carol Forsblom, Valma Harjutsalo, Per-Henrik Groop

**Affiliations:** 1 Folkhälsan Institute of Genetics, Folkhälsan Research Center, Helsinki, Finland; 2 Abdominal Center Nephrology, University of Helsinki and Helsinki University Central Hospital, Helsinki, Finland; 3 Research Program for Clinical and Molecular Metabolism, Faculty of Medicine, University of Helsinki, Helsinki, Finland; 4 Diabetes Prevention Unit, National Institute for Health and Welfare, Helsinki, Finland; 5 Department of Diabetes, Central Clinical School, Monash University, Melbourne, Victoria Australia

**Keywords:** Type 1 diabetes, Risk factors

## Abstract

We assessed meal timing, meal frequency, and breakfast consumption habits of adult individuals with type 1 diabetes (n = 1007) taking part in the Finnish Diabetic Nephropathy Study, and studied whether they are associated with glycaemic control. Data on dietary intake and blood glucose measurements were retrieved from food records. HbA_1c_ was measured at the study visit. In the whole sample, four peaks of energy intake emerged. Energy intake was the greatest in the evening, followed by midday. Altogether 7% of the participants reported no energy intake between 05:00 and 09:59 (breakfast skippers). While breakfast skippers reported lower number of meals, no difference was observed in the total energy intake between those eating and omitting breakfast. In a multivariable model, skipping breakfast was associated with higher mean blood glucose concentrations and lower odds of good glycaemic control. A median of 6 daily meals was reported. Adjusted for confounders, the number of meals was negatively associated with HbA_1c_, and the mean of the blood glucose measurements, but positively associated with the variability of these measurements. Our observations support the habit of a regular meal pattern, including consumption of breakfast and multiple smaller meals for good glycaemic control in adults with type 1 diabetes. However, an increase in the blood glucose variability may additionally be expected with an increase in the number of meals eaten.

## Introduction

For adults with diabetes healthy eating patterns, with an emphasis on a variety of nutrient-dense foods in appropriate portion sizes, are recommended^1^. While such a “healthy eating pattern” is expected to address individual nutritional needs and take into account personal and cultural preferences, not much is said about meal frequencies or distribution of energy intake throughout the day. Important to individuals with diabetes, recent evidence suggests that both meal frequency and circadian energy distribution can influence glycaemic control.

In studies of meal frequency, conducted in individuals with type 1 diabetes, a meal pattern with smaller and more frequent meals have been associated with better glycaemic control^2–4^. Similarly, interventional trials in women with polycystic ovary syndrome^5^ and in people with impaired glucose tolerance or overt type 2 diabetes^6^, comparing eating patterns with 3 and 6 daily meals have shown more favourable levels of glycaemia with higher meal frequencies. On the other hand, Thomsen *et al*. detected no between-treatment differences in insulin sensitivity or the glucose and insulin responses to a test meal following interventions with 3 and 8 daily meals in type 2 diabetes^7^. In line with the observations by Thomsen *et al*., meal frequency was not associated with the risk of incident type 2 diabetes in women participating in the Nurses’ Health Study^8^. Moreover, in lean healthy men, extremely high eating frequency (3 vs. 14) resulted in significantly higher 24-hour glucose concentration^9^.

Omitting breakfast is traditionally considered an unhealthy habit. In support of this, participants in the Nurses’ Health Study with irregular breakfast habits had increased risk of incident type 2 diabetes compared with those who regularly consumed breakfast^8^. In another study of individuals with type 2 diabetes, skipping breakfast was associated with an almost 11% increase in HbA_1c_^10^. Moreover, scheduling higher energy intake in the mornings, as compared with evenings, led to overall reduced glucose excursions throughout the day in type 2 diabetes^11^. While studies in type 1 diabetes are scarce, there is some evidence from adolescents with type 1 diabetes, that eating breakfast is associated with better glycaemic control^2^.

Not much is known about the circadian eating pattern of adult individuals with type 1 diabetes and how meal frequency and breakfast skipping are related to glycaemic control. Our aim was, therefore, twofold. First, we elucidated the distribution of energy and macronutrient intakes over the course of the day in a large sample of individuals with type 1 diabetes. Second, we described the meal frequencies and breakfast skipping habits in this population and investigated whether they were associated with glycaemic control.

## Results

### Eating pattern of the whole sample

**Figure 1:**
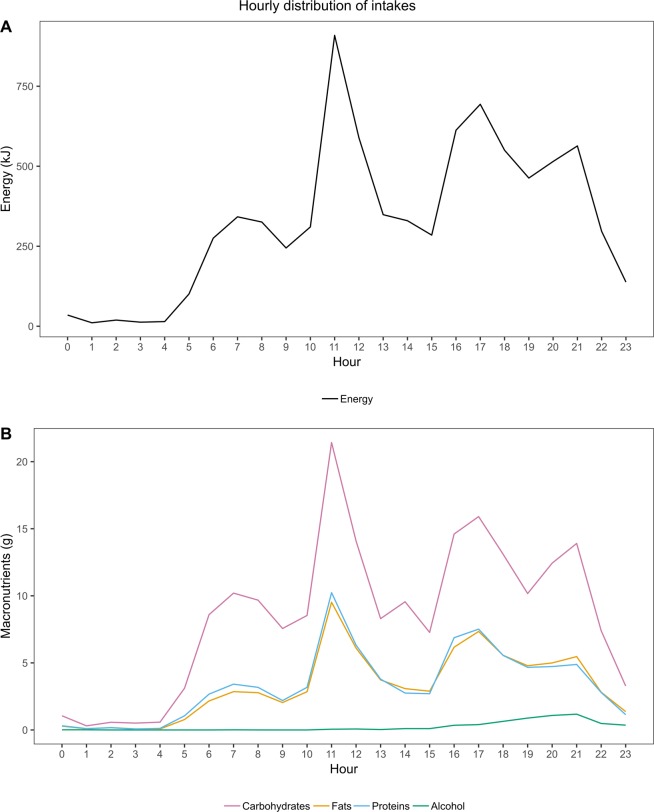
Hourly distributions of energy and macronutrient intakes of the whole sample. (**A**) Energy intake in the whole sample. (**B**) Macronutrient intakes in the whole sample.

Data were available from a total of 1007 individuals (41% men, median age 47 years) with type 1 diabetes (Table 1). Approximately one third of the sample achieved good glycaemic control, as defined by HbA_1c_ below 59 mmol/mol (<7.5%). The mean reported daily energy intake of the sample was 7988 kJ (Table 2). The highest energy intake, 40.4% of the total energy intake, took place between 17:00 and 23:59. This was followed by midday with 28.7%, morning with 16.1%, afternoon with 13.6%, and night with 1.2% of the total energy intake. Calculated by hourly intake at each of the periods, the largest energy intake was observed at midday, followed by evening, afternoon, morning, and night. The distribution of energy intake for the entire sample during the whole day revealed four peaks timed at around 06:00–08:00, 11:00, 16:00–17:00, and 21:00 (Fig. 1A).

**Table 1:** Participant characteristics.

	n = 1007
Number of meals	6 (5–8)
Men, %	40.6
Age, years	47 (36–57)
Diabetes duration, years	30 (20–41)
Current smoking, %	10.6
Insulin pump, %	18.5
BMI, kg/m^2^	25.5 (23.1–28.4)
Weight, kg	75 (65–84)
HbA_1c_, mmol/mol	63 (55–72)
HbA_1c_, %	7.9 (7.2–8.7)
HbA_1c_ < 59 mmol/mol (<7.5%), %	33.1
Mean of the BG measurements, mmol/l	8.1 (6.9–9.4)
CV of the BG measurements	0.4 (0.3–0.5)
Triglycerides, mmol/l	0.97 (0.74–1.36)
Total cholesterol, mmol/l	4.5 (3.9–5.1)
HDL cholesterol, mmol/l	1.6 (1.3–1.9)
Systolic blood pressure, mmHg	136 (124–150)
Diastolic blood pressure, mmHg	76 (70–83)

Data are presented as frequency (%) or median (interquartile range). BMI, body mass index; BG, blood glucose; CV, coefficient of variation.

**Table 2:** Energy and macronutrient intakes at different times of the day.

	Energy kJ/hour	Energy kJ	E%	CHO G	% of CHO	Fat g	% of fat	Protein g	% of PRO	Alcohol g	% of ALC
00:00–04:59	18 ± 2	92 ± 12	1.2	3.0 ± 0.4	1.5	0.7 ± 0.1	0.9	0.8 ± 0.1	1.0	0.02 ± 0.02	0.4
05:00–09:59	258 ± 5	1289 ± 25	16.1	39.2 ± 0.8	19.4	10.6 ± 0.3	13.6	12.5 ± 0.3	15.6	0.01 ± 0.01	0.2
10:00–14:00	574 ± 10	2295 ± 39	28.7	56.3 ± 1.0	27.8	23.5 ± 0.5	30.1	24.7 ± 0.5	30.7	0.19 ± 0.05	3.3
14:01–16:59	363 ± 13	1088 ± 39	13.6	27.4 ± 1.0	13.6	10.8 ± 0.5	13.9	11.1 ± 0.5	13.8	0.52 ± 0.13	9.0
17:00–23:59	461 ± 8	3224 ± 57	40.4	76.3 ± 1.4	37.7	32.4 ± 0.7	41.5	31.3 ± 0.7	38.9	5.02 ± 0.47	87.1
24 hours	333 ± 3	7988 ± 79	100.0	202.2 ± 2.4	100.0	78.0 ± 1.0	100.0	80.4 ± 1.0	100.0	5.77 ± 0.50	100.0

Data are presented as mean ± SE or percentage of total energy. E%, percentage of total energy; CHO, carbohydrates; PRO, proteins; ALC, alcohol.

As seen for the energy intake, the largest amounts of carbohydrates, fats, proteins, and alcohol were consumed in the evening hours (Table 2). For carbohydrates, fats, and proteins, meals eaten at midday contributed the second largest portion of the total daily intakes. Morning represented the time with the third largest intakes of carbohydrates and proteins. For fats, instead, the third largest intake took place in the afternoon. Followed by the evening hours, most of the remaining alcohol was consumed in the afternoon and midday. In the morning and night hours, instead, the mean alcohol intake was negligible. The hourly distributions of the macronutrients revealed that fat and protein intakes exhibited four peaks similar to those seen for energy intake (Fig. 1B). For carbohydrate intake, instead, a fifth smaller peak emerged at 14:00, suggesting an afternoon snack of relatively high carbohydrate content. Alcohol intake showed a gradual increase from late afternoon onwards, with the highest intakes at 21:00.

### Breakfast skipping

**Figure 2:**
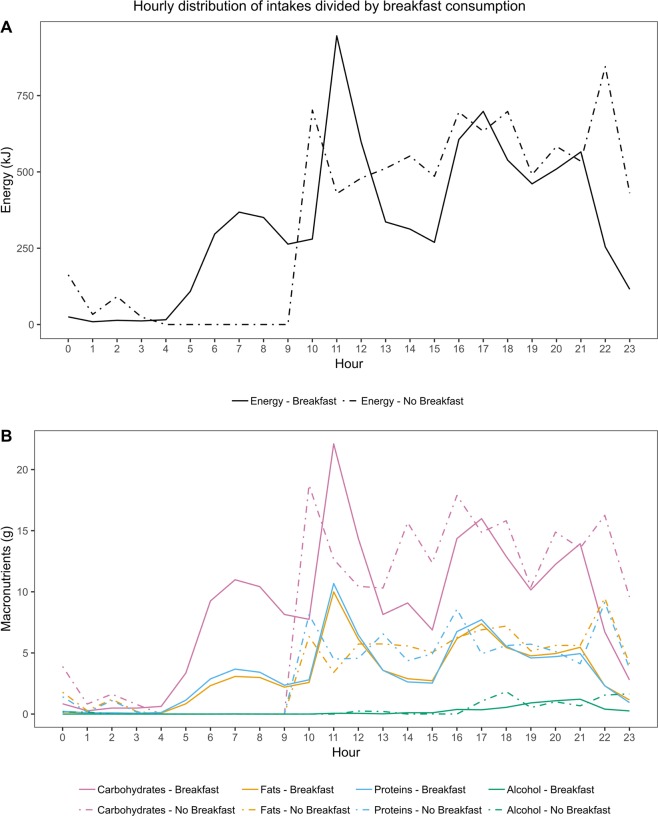
Hourly distributions of energy and macronutrient intakes divided by breakfast consumption. (**A**) Energy intake by breakfast consumption. (**B**) Macronutrient intakes by breakfast consumption.

A total of 72 individuals (7%) reported no dietary intake between 05:00 and 09:59 and were thus defined as those skipping breakfast. Individuals skipping breakfast were younger with shorter diabetes duration, and they had a higher mean reported blood glucose concentration, as compared to those who reported eating breakfast (Table 3). Although not significant (p = 0.051), there was also a tendency for the breakfast skippers to less frequently reach the glycaemic goal of HbA_1c_ < 59 mmol/mol (<7.5%). While the total energy intakes between the two groups were comparable, those skipping breakfast reported higher energy intake at night, afternoon, and evening (Table 4). Visual inspection of the energy distribution throughout the day suggested that those skipping breakfast had relatively high energy intake during midday, afternoon, and evening, with multiple but shallower peaks (Fig. 2A). Moreover, compared to those eating breakfast, energy, carbohydrate, fat, and protein intakes peaked at an earlier hour in the midday, and at a later hour in the evening (Fig. 2A,B).

**Table 3:** Participant characteristics divided by breakfast consumption.

	Breakfast n = 935 (93%)	No breakfast n = 72 (7%)	p
Number of meals	6 (5–8)	5 (4–7)	<0.001
Men, %	39.9	50.0	0.105
Age, years	48 (37–58)	38 (29–47)	<0.001
Diabetes duration, years	31 (20–42)	20 (12–35)	<0.001
Current smoking, %	10.7	10.1	1.000
Insulin pump, %	17.8	27.1	0.057
BMI, kg/m^2^	25.5 (23.2–28.3)	25.6 (22.8–29.1)	0.986
Weight, kg	74 (65–84)	78 (67–90)	0.089
HbA_1c_, mmol/mol	63 (55–72)	64 (59–74)	0.367
HbA_1c_, %	7.9 (7.2–8.7)	8.0 (7.5–8.9)	0.367
HbA_1c_ < 59 mmol/mol (<7.5%), %	33.9	22.2	0.051
Mean BG measurements, mmol/l	8.0 (6.8–9.3)	9.2 (7.7–10.1)	<0.001
CV of the BG measurements	0.4 (0.3–0.5)	0.4 (0.3–0.5)	0.932
Triglycerides, mmol/l	0.96 (0.73–1.33)	1.10 (0.80–1.48)	0.067
Total cholesterol, mmol/l	4.5 (3.9–5.1)	4.6 (4.1–5.2)	0.175
HDL cholesterol, mmol/l	1.6 (1.3–1.9)	1.5 (1.3–1.8)	0.120
Systolic blood pressure, mmHg	136 (124–150)	132 (122–149)	0.501
Diastolic blood pressure, mmHg	76 (69–83)	76 (72–83)	0.470

Data are presented as frequency (%) or median (interquartile range). BMI, body mass index; BG, blood glucose; CV, coefficient of variation.

**Table 4:** Energy and macronutrient intakes at different times of day divided by breakfast consumption status.

	Energy (kJ) Breakfast	No BF	CHO (g) Breakfast	No BF	Fat (g) Breakfast	No BF	Protein (g) Breakfast	No BF	Alcohol (g) Breakfast	No BF
00:00–04:59	75 ± 10	312 ± 107^b^	2.7 ± 0.4	7.1 ± 2.2^b^	0.5 ± 0.1	3.5 ± 1.5^b^	0.6 ± 0.1	2.8 ± 1.1^b^	0	0.32 ± 0.32^c^
05:00–09:59	1389 ± 24	0^c^	42.2 ± 0.8	0^c^	11.4 ± 0.3	0^c^	13.5 ± 0.3	0^c^	0.01 ± 0.01	0
10:00–14:00	2295 ± 39	2292 ± 189	56.3 ± 1.1	55.7 ± 4.7	23.5 ± 0.6	23.3 ± 2.4	24.7 ± 0.5	25.4 ± 2.8	0.17 ± 0.05	0.45 ± 0.32
14:01–16:59	1051 ± 39	1566 ± 175^b^	26.3 ± 1.0	42.4 ± 5.0^b^	10.5 ± 0.5	14.8 ± 2.0^a^	10.7 ± 0.5	16.2 ± 2.1^b^	0.56 ± 0.14	0.01 ± 0.01
17:00–23:59	3147 ± 57	4216 ± 295^c^	74.8 ± 1.4	95.4 ± 7.8^a^	31.5 ± 0.7	44.1 ± 3.9^b^	30.8 ± 0.7	38.4 ± 3.4	4.77 ± 0.47	8.30 ± 2.35
24 hours	7957 ± 81	8386 ± 337	202.3 ± 2.5	201.5 ± 10.4	77.4 ± 1.1	85.6 ± 4.7	80.2 ± 1.0	82.8 ± 4.5	5.51 ± 0.51	9.08 ± 2.37

CHO, carbohydrate; BF, breakfast.

^a^p < 0.05, ^b^p < 0.01, ^c^p < 0.001.

We then investigated whether skipping breakfast was associated with glycaemic control. Adjusted for sex, diabetes duration, smoking, energy intake, physical activity and mode of insulin administration, we observed that breakfast skipping was associated with reduced odds of achieving good glycaemic control (Table 5). Moreover, skipping breakfast was associated with higher mean of the reported blood glucose measurements (adjusted means 8.8 ± 0.2 mmol/l vs. 8.2 ± 0.1 mmol/l, p = 0.023).

**Table 5:** Associations between breakfast consumption, meal frequency and glycaemic control.

	Adjusted means		B	95% CI	p
	Breakfast	No BF			
HbA_1c_ < 59 mmol/mol (<7.5%)			0.510	0.262–0.994	0.048
HbA_1c_	64.1 ± 0.5	66 ± 1.7	1.447	−2.090–4.983	0.423
Mean of the BG measurements	8.2 ± 0.1	8.8 ± 0.2	0.609	0.105–1.112	0.018
CV of the BG measurements	0.41 ± 0.01	0.43 ± 0.02	0.019	−0.015–0.053	0.280
	≥8 meals	≤5 meals			
HbA_1c_ < 59 mmol/mol (<7.5%)			0.741	0.472–1.164	0.194
HbA_1c_	62 ± 1.0	66 ± 0.9	3.944	1.254–6.633	0.004
Mean of the BG measurements	8.0 ± 0.1	8.6 ± 0.1	0.588	0.209–0.967	0.002
CV of the BG measurements	0.42 ± 0.01	0.40 ± 0.01	−0.022	−0.049–0.005	0.113

Models are adjusted for sex, diabetes duration, smoking, energy intake, physical activity, and mode of insulin administration. Logistic regression analysis for the dichotomised variable (HbA_1c_ < 59 mmol/mol), and generalised linear regression analysis for the continuous variables. BF, breakfast; BG, blood glucose; CV, coefficient of variation. In the logistic regression analyses, breakfast = 0, no breakfast = 1; ≥ 8 meals = 0, ≤ 5 meals = 1.

### Number of meals and glycaemia

The number of reported meals ranged from 3 to 20, with a median of 6 (interquartile range from 5 to 8). Adjusted for sex, diabetes duration, smoking, energy intake, physical activity and mode of insulin administration, the number of meals was negatively associated with HbA_1c_ (B, 95% Wald Confidence Interval, −0.615, −1.108 to −0.123, p = 0.014), and the mean of the blood glucose measurements (−0.097, −0.166 to −0.028, p = 0.006), but positively associated with the variability of the blood glucose measurements (0.006, 0.001 to 0.010, p = 0.020). Compared with individuals in the highest quartile based on the number of meals (≥8 meals), those in the lowest quartile (≤5 meals) had significantly higher HbA_1c_ and mean of the blood glucose measurements (Table 5).

## Discussion

In this sample of Finnish adults with type 1 diabetes, a circadian eating pattern with four major peaks of energy intake, timed at breakfast, lunch, dinner, and evening meal, emerged. While protein and fat intakes mirrored that of total energy intake, an additional smaller peak of carbohydrate intake was observed in the afternoon. Alcohol intake was most pronounced in the evening hours. Altogether, 7% of the sample omitted breakfast. The overall circadian profile of the energy intake in those skipping breakfast differed significantly from those reporting energy intake in the morning hours. Of interest, the mean energy intake of the breakfast skippers remained at high levels throughout the rest of the day, leading to a total energy intake comparable to those who reported eating breakfast. Importantly, breakfast skipping was associated with higher mean values of daily blood glucose measurements and lower odds of reaching good glycaemic control. A median of 6 daily meals was reported in the current study. Higher number of reported meals was associated with higher variability of the blood glucose measurements but better glycaemic control, measured as mean of the reported blood glucose concentrations and HbA_1c_.

The question regarding the association between meal frequency and glycaemia has been addressed in a number of epidemiological studies among individuals with type 1 diabetes. In one such study of adolescents with type 1 diabetes, similarly to the current observations, the reported number of meals was associated with lower HbA_1c_^2^. Yet in another sample of adolescents with type 1 diabetes, higher number of eating occasions was associated with better glycaemic control and, together with BMI, social class, and day-to-day variation in energy intake, explained 38% of the variation in the mean HbA_1c_ values of the preceding year^3^. Øverby *et al*. investigated the dietary practices of 655 children and adolescents with type 1 diabetes^4^. In their analyses, those who skipped meals were observed to have higher odds of suboptimal HbA_1c_. Among the 687 participants in the intensive treatment arm of the Diabetes Control and Complications Trial, instead, together with adherence to the prescribed diet, prompt treatment of hyperglycaemia, and avoidance of overtreatment of hypoglycaemia, avoidance of extra snacks appeared beneficial^12^.

Along with the epidemiological studies of meal frequency and glycaemia, the question has also been addressed in a number of interventional trials. In these trials, energy intake is typically kept constant with the number of meals being the only difference between the treatments. In one such study, 15 normal-weight middle-aged men and women underwent two 8-week diet interventions during which they consumed all the energy for weight maintenance in either 1 or 3 daily meals^13^. In a randomised cross-over design, the three meals were consumed at breakfast, lunch, and dinner, while in the one meal plan all foods were eaten during a four-hour time period in the early evening hours. During the one meal dietary regimen, morning plasma glucose concentrations were significantly increased. Moreover, while fasting plasma insulin concentrations were not affected, the less frequent meal plan resulted in worse glucose tolerance as indicated by significantly greater and more prolonged elevation of plasma glucose concentrations. Of importance, the detrimental effects on the glucose tolerance brought about by the one meal per day pattern were rapidly reversed upon returning to the thrice a day meal frequency, indicating that the diet caused no long-lasting effects on glucose metabolism. In another study of 40 weight-stable women with polycystic ovary syndrome a 6-meal pattern significantly improved insulin sensitivity compared to a 3-meal pattern^5^. Yet in another study among healthy lean men, two isoenergetic diets were consumed either over 3 or 14 daily eating occasions^9^. In that study, the area under curve of the 24-hour glucose concentration was lower during the 3-meal plan, suggesting that extremely high eating frequencies may not be of additional benefit. In another study of multiple meals, however, mean blood glucose concentrations of healthy men were no different during interventions with 3 and 17 daily meals^14^. In the same study, during the nibbling diet a 28% decrease in the serum insulin level was observed. While we were not able to identify any interventions involving subjects with type 1 diabetes, a number of trials have been conducted among individuals with type 2 diabetes. In one such study, for a duration of two weeks, the daily energy was consumed in random order as either three or eight meals^7^. In that study, different meal frequencies were not associated with insulin sensitivity or the glucose and insulin responses to a high-carbohydrate test meal at the end of the intervention. In another randomised crossover trial, individuals with type 2 diabetes followed a 12-week weight-maintenance diet with either 3 or 6 daily meals^6^. Here, compared to the less frequent meal plan, following the 6-meal plan significantly reduced HbA_1c_ and plasma glucose concentrations at 120 minutes post oral glucose tolerance test. Finally, following an overnight fast, 12 individuals with type 2 diabetes were assigned in random order to two 8-hour observation periods^15^. During these periods, isoenergetic diets as either two or six meals were consumed. Although, during the study period, there was no difference in the incremental blood glucose area between the interventions, the postprandial blood glucose fluctuations, insulin, and free fatty acid concentrations were lower with increasing meal frequency.

While there are differences in the methods used and populations investigated, in the studies described above, a cautious conclusion may be drawn that dividing the daily energy intake into multiple smaller meals may be of some benefit for individuals with type 1 diabetes. Our observation showing that a higher number of meals was associated with better glycaemic control is in support of this conclusion. A number of phenomena may explain the benefit of dividing energy intake throughout the day. First, spreading the nutrients into smaller meals could reduce the impact of glycaemic load at individual meals^16^. Second, distributing the total daily energy into multiple meals may be of benefit to individuals administering external insulin, as estimating carbohydrate content of the smaller meals is easier^17^. Finally, the elevated free fatty acid levels related to the increase in meal spacing is known to impact glucose metabolism by reducing insulin-mediated glucose disposal in the muscle, stimulating gluconeogenesis, and increasing hepatic glucose output. While it is widely acknowledged that good glycaemic control is an important factor for the long-term vascular health, large variability of the blood glucose concentrations may additionally play a role in the pathology of end-organ damage in diabetes^18^. Therefore, identifying factors related to the blood glucose variability could be of importance. Of interest higher number of meals, in the current study, was additionally associated with increased variability of the blood glucose measurements. It has to be acknowledged, however, that no data on hypoglycaemia episodes were available for the current analyses. Moreover, we did not identify indications for food intake. Indeed, it is highly probable that a number of eating occasions took place in order to treat hypoglycaemia. Therefore, the observations related to the association between the number of meals and increased blood glucose variability could reflect the need to correct low blood glucose values upon experiencing hypoglycaemia.

To the best of our knowledge, the current study is amongst the first ones to describe the circadian energy intake and breakfast habits of adult individuals with type 1 diabetes. From a previously published study of adolescents with type 1 diabetes we learned, however, that 74% of girls and 98% of boys daily consumed breakfast^2^. Lunch and dinner were reported by 48% and 90% of the sample, respectively, with no differences between the sexes. Evident in girls, skipping breakfast and lunch were both associated with worse glycaemic control, while breakfast omission was additionally associated with higher eating disorder psychopathology including insulin omission due to weight concerns. From the previously published studies of non-type-1-diabetes populations we additionally learned that in a representative sample of the US adult population nearly 19% omitted breakfast^19^. In a Finnish population-based survey, instead, 97% of men and 96% of women reported eating breakfast^20^. Finally, in a small sample of non-shift-working individuals with type 2 diabetes, approximately 11% self-reportedly omitted breakfast^10^. The current observation of 7% of individuals skipping breakfast falls within the results from these previous reports. Similar to the previous observations^21^, breakfast omission in the current study was associated with a lower total number of meals. However, as the energy intake of those reporting and not reporting eating breakfast was comparable, those omitting breakfast compensated for the missed morning energy intake during the subsequent eating occasions, suggesting a pattern of larger but fewer meals. Subsequently, compared to the breakfast eaters who, beyond breakfast, exhibited three major peaks of energy intake, the curve describing the mean energy intake in those omitting breakfast had multiple but smaller peaks from 10:00 o’clock onwards. Given the lower median meal frequency, this observation suggests that the breakfast skippers of the current study are, amongst themselves, quite heterogeneous in their meal timings.

The association between breakfast consumption and glycaemic fate has been investigated in various populations. In the Health Professionals Follow-Up Study, for example, eating patterns of 29,206 US men were assessed and participants were followed-up for 16 years^22^. Over that period, 1944 incident cases of type 2 diabetes were identified. Adjusted for known risk factors for type 2 diabetes, men skipping breakfast had 21% increased risk of type 2 diabetes, compared with men who reported eating breakfast. Similarly, in the Nurses’ Health Study, compared to those consuming breakfast 7 times per week, the risk of type 2 diabetes was significantly higher in those who consumed breakfast irregularly (≤6 times per week)^8^. Yet, in another observational study among individuals with type 2 diabetes, skipping breakfast was associated with almost 11% higher HbA_1c_ values^10^. Amongst the clinical trials, in this field, is a randomised controlled trial by Betts *et al*., who investigated the role of breakfast in healthy lean adults^23^. In their study, individuals fasting until noon for a 6-week period experienced increased glycaemic variability during afternoons and evenings. Instead, the practice of regularly consuming breakfast helped to maintain more stable blood glucose responses. In another study with a randomised crossover design, Kobayashi *et al*. assessed the blood glucose concentrations of eight young men in two experimental conditions^24^. In one of the conditions, participants ate breakfast, lunch, and dinner, while in the other condition the same amount of energy was consumed at lunch and dinner times only. Skipping breakfast increased the average blood glucose concentration during afternoon and sleep, subsequently resulting in overall increased 24-hour average blood glucose concentration. Nas *et al*. investigated the glucose metabolism of healthy adults in conditions of breakfast skipping and dinner skipping^25^. In their study, compared to omitting dinner, breakfast skipping resulted in higher glucose concentrations and insulin resistance after lunch. Moreover, the observed increase in post-lunch fat oxidation, occurring despite of increased insulin concentration, was suggestive of metabolic inflexibility after prolonged fasting. Also, in type 2 diabetes, omission of breakfast was associated with an increased glycaemic response after lunch and dinner, when compared to the glycaemic responses taking place after lunch and dinner when breakfast was consumed^26^.

Observations related to the breakfast omission, subsequent larger meals timed at later hours, and compromised glycaemic control may be related to a decrease in insulin sensitivity and glucose tolerance towards evening. Indeed, independent of the behavioural cycle, postprandial blood glucose concentrations are significantly higher in the evening compared to the morning^27^. Moreover, circadian misalignment by 12 hours tend to increase the postprandial glucose concentrations^27^. Of note, the above variations in glucose tolerance seemed to be explained by different mechanisms. The decreased pancreatic beta cell function was effective during the evening, while decreased insulin sensitivity seemed to be behind the effect during circadian misalignment. In line with the above observations, compared with a high-energy dinner, a high-energy breakfast resulted in greater improvements in fasting glucose, insulin, and insulin resistance, despite an overall similar energy intake throughout the day^28^. Moreover, following the lunch of similar energy contents, serum glucose and insulin responses were significantly lower when the high-energy breakfast was consumed. The above described reduced glycaemia related to a meal consumed after breakfast is known as the second meal phenomenon. This phenomenon could be due to a breakfast-induced increase in beta cell responsiveness seen during the second meal. Here, beta cell memory and the magnitude of insulin release is enhanced by the earlier glucose exposure. Of importance, the second meal phenomenon is not restricted to the post-breakfast lunch, but seems to persist throughout the day as breakfast omission not only worsened the postprandial glucose and impaired insulin secretion at lunch, but also at dinner^26^.

We acknowledge that the current study was observational in nature and applied dietary data collected using a self-report method. While some reservations may be related to the self-reported energy intake, we are not aware if the self-report method impacts the reporting of meals and their timings. In the current analyses, data from only one day per participant were used. Whether the selected day is representative of the dietary practices of the participants at large, is not known. HbA_1c_ was measured at the study visit, and was therefore measured prior to the dietary assessment. In case the reported dietary practices were not representative of the typical diet, the results relying on the reverse assessments of HbA_1c_ and dietary exposure could be biased. The blood glucose measurements were, however, conducted at the time of the dietary assessment. A large study sample of well-characterised individuals and the use of a record instead of a memory-relying recall method to collect data on dietary intake and blood glucose measurements are also considered strengths of this study.

In conclusion, large variation in meal frequencies was observed in this sample of adult individuals with type 1 diabetes. Despite this, a pattern of 4 major peaks of energy intake was evident in the whole population. Individuals reporting and not reporting eating breakfast had comparable total energy intakes but distinctive patterns of circadian distribution of dietary energy. Finally, our observations support the practice of a regular meal pattern, with breakfast and multiple smaller daily meals for better glycaemic control.

## Methods

Study subjects were participants of the Finnish Diabetic Nephropathy (FinnDiane) Study. Type 1 diabetes was defined as diabetes onset before the age of 40 years, and permanent insulin treatment initiated within a year from the diagnosis. The Ethics Committee of The Helsinki and Uusimaa Hospital District approved the study protocol. The study was carried out in accordance with the relevant guidelines and regulations. Written informed consent was obtained from all individuals prior to study participation.

At the FinnDiane Study visit, participants were thoroughly examined. This included measurements of height, weight, and blood pressure. From these measurements body mass index (BMI; kg/m^2^) and the mean of two blood pressure measurements were calculated. Non-fasting, early morning blood samples were collected and sent to a central laboratory to measure serum lipid and lipoprotein concentrations. HbA_1c_ was measured at each study site using a photometric, enzymatic assay. Smoking was self-reported, and those reporting current smoking were identified for the analyses. Mode of insulin administration was self-reported. Physical activity was assessed using a questionnaire on leisure-time physical activity as previously described^29^. Here, for the preceding 12 months, mean frequency, single session duration, and intensity of 21 common forms of leisure-time activities were assessed. In order to calculate the physical activity as the metabolic equivalent of task hours (METh), the activity- and intensity-specific metabolic equivalents were multiplied by the duration of the activity. The METh was used as a continuous variable in the analyses.

Two methods were used to assess dietary intake, as described by Ahola *et al*.^30^. First, participants completed a diet questionnaire, which gives an overall picture of the participants’ dietary practices. In the questionnaire, the participants report their customary consumption habits of tea, coffee, liquid milk products, breads, spreads, cooking fats, salt, probiotics containing foodstuffs and dietary supplements. Additionally, adherence to any special diets and to the dietary recommendations provided by the health-care personnel were queried. Included was also a 19-item food frequency questionnaire where, on a 7-level scale, the consumption frequencies of fish dishes, meat dishes, poultry, sausages and cold cuts, eggs, legumes, fresh vegetables, cooked vegetables, potatos, pasta and rice, fruits and berries, fatty cheese, low-fat cheese, yoghurt and curd, ice cream, soft drinks, sweet pastries, sweets and chocolates, and fried and grilled foods were reported. The questionnaire has previously been validated in the FinnDiane Study population of participants with type 1 diabetes^31^. Upon returning the diet questionnaire, participants were sent an allocated 3-day diet record covering two weekdays and one weekend day (consecutive Sunday-Tuesday or Thursday-Saturday). A second 3-day record (Thursday-Saturday or Sunday-Tuesday, respectively) was completed within 2 to 3 months. In the current study, only data collected with the diet record was used. From the completed records, we investigated the dietary intake as reported on the second day of recording. The second day was selected over the first one to ensure that participants had also included night-time eating in the records. Compared to the later days, which may have been burdened by increasing exhaustion related to the record-keeping, the second day was thought to better represent the habitual dietary intake. To investigate the energy and macronutrient intakes over the course of the day, we formed five periods as follows: night (from 00:00 to 04:59), morning (from 05:00 to 09:59), midday (from 10:00 to 13:59), afternoon (from 14:00 to 16:59), and evening (from 17:00 to 23:59), modified from the paper by Mathias *et al*.^32^. As these periods differed in their duration, energy intake per hour was also calculated for each of the five periods. In addition, energy and macronutrient intakes per hour (e.g. intakes reported between 10:00 and 10:59 contributed towards the hour “10:00”) were used to plot the intakes in a graph. AivoDiet software (version 2.0.2.3, AIVO, Turku, Finland) was used to calculate energy and macronutrient intakes. Data from individuals with reported energy intake between 3347 and 14644 kJ (800 and 3500 kcal) were included. When choosing the cut-off values, we took note of Willet´s publication stating that mean daily energy intakes between 500 and 4000 kcal may be considered appropriate^33^. However, upon further considerations based on the characteristics of the current study population (type 1 diabetes, mean age and BMI), we chose to use these more conservative cut-off values.

In addition, in order to investigate the circadian energy and macronutrient intakes of the whole population, we formed two groups based on the reported energy intake in the morning hours. Those with no reported energy intake between 05:00 and 09:59 were considered breakfast skippers, and their energy and macronutrient intakes were compared to those of the remaining sample. From the diet record entries, we also calculated the number of reported meals. Here, each report of consuming any energy-containing foodstuffs with a distinct eating time was considered as a meal. In the analyses, we investigated the association between the eating patterns (breakfast skipping and meal frequency) and glycaemic control. As measures of glycaemic control, we included HbA_1c_ as a continuous variable, HbA_1c_ as a dichotomised variable (values < 59 mmol/mol [7.5%] representing “good glycaemic control”), and the mean and coefficient of variation of the reported daily blood glucose measurements. Data for the two latter variables were obtained from the diet records as, in addition to reporting their food intake, the participants had also reported the results of their blood glucose measurements.

### Statistical analyses

Data concerning the study population are presented as frequencies for categorical variables, and median (interquartile range) for continuous variables with skewed distribution. Although, many dietary variables also exhibited skewed distribution, we chose to present these data as means ± standard errors, instead. This was done because, for a number of these variables, the median for the whole population was zero, which was not considered particularly informative. Between-group comparisons were conducted with Chi squared test for categorical variables, and Mann-Whitney U-test for continuous variables. Logistic regression analysis and generalized linear regression were used for the respective multivariable analyses. For the analyses, IBM SPSS Statistics for Windows, Version 22.0 (IBM Corp, Armonk, NY, USA) was used. A two-tailed *P* value < 0.05 denoted statistical significance.

## Supplementary information

Supplementary information 
